# Serum homocysteine level in vegetarians in District Tharparker, Sindh

**DOI:** 10.12669/pjms.311.6111

**Published:** 2015

**Authors:** Aneel Kapoor, Nudrat Anwar Zuberi, M. Imran Rathore, Mukhtiar Baig

**Affiliations:** 1 Aneel Kapoor, MPhil, Assistant Professor, Department of Biochemistry, Muhammad Medical College, Mirpurkhas, Sind, Pakistan.; 2 Nudrat Anwar Zuberi, PhD, Professor & Head of Biochemistry Department, BMSI, JPMC, Karachi, Pakistan.; 3 Muhammad Imran Rathore, MPhil, Department of Anatomy, Muhammad Medical College, Mirpurkhas, Sind, Pakistan.; 4 Dr Mukhtiar Baig, PhD, MHPE, Professor of Clinical Biochemistry & Head of Assessment Unit, Faculty of Medicine, Rabigh, King Abdulaziz University, Jeddah- 21577, Kingdom of Saudi Arabia.

**Keywords:** Homocysteine, Vegetarians, Omnivores

## Abstract

**Objectives:**

The aim of present study was to investigate serum homocysteine levels in apparently healthy vegetarians and ominvores in Mithi, district Tharparker, Sindh, Pakistan.

**Methods:**

This study was conducted in the Department of Biochemistry, Basic Medical Sciences Institute (BMSI), Jinnah Postgraduate Medical Center (JPMC), Karachi and blood samples were collected from Mithi, district Tharparker, Sindh, Pakistan, in 2012. One hundred vegetarian and one hundred omnivores (age ranging from 20-40 years) were enrolled for this study. Serum homocysteine levels were measured by the chemiluminescence enzyme immunoassay method.

**Results:**

Serum homocysteine (Hcy) level was considerably higher (p<0.001) in vegetarian group compared to omnivores. We further grouped and analyzed our study subjects according to their gender and according to Hcy level (greater than or lower than 15µmol/L). A considerable number of vegetarian subjects 30% were having Hcy >15µmol/L compared to omnivores 6%, (p<0.001). Gender-wise comparison showed that 27.02% male and 38.46% females had >15µmol/L serum Hcy level in vegetarian group and 6.9% male and 3.5% females had >15µmol/L serum Hcy level in omnivores group, but the difference was not significant in any group.

**Conclusion:**

Vegetarians are more prone to develop hyperhomocysteinemia, so they are at high risk to develop cardiovascular disease.

## INTRODUCTION

Cardiovascular disease (CVD) is the most common cause of death in the world. Several risk factors are associated with CVD like increased low density lipoprotein levels, low levels of high density lipoproteins, high blood pressure, obesity and diabetes mellitus.^1^ Elevated homocystein (Hcy) levels have also been recognized as an independent risk factor for CVD.^2^^-^^4^ In addition, it is demonstrated that each 5 mmol/l increase in plasma tHcy is linked with around 20 % enhance threat of CHD events. Elevated Hcy levels facilitate thrombogenesis, damage endothelial function, and support lipid perioxidation and smooth muscle proliferation. Especially in South Asian population, CVDs rates are very high, and in Pakistan it has been reported that CVD results in more than 100,000 casualties’ annually.^5^

Homocysteine is an unstable amino acid, which is metabolized either by the remethylation pathway to methionine or the trans-sulfuration pathway to cysteine. Elevated Hcy levels causes increased production of free oxygen radicals and an oxidative stress, and this contributes to atherosclerosis.^6^

The normal homocysteine (tHcy) level ranges from 5 to 15µmol/L while its level greater than >15 µmol/l is considered as Hyperhomocysteinemia. There are various reasons which may cause elevation in homocystein level like genetic defects, certain drugs, renal insufficiency, or deficiencies of vitamin B6, folate, or vitamin B12.^7^ Shridhar et al.,^8^ has pointed out significantly decrease intake of vitamin B12 in Indian vegetarian diets which is one of the most important causes of elevated level of homocysteine. Several studies have reported significantly higher level of homocysteine in vegetarians than those in omnivores.^9^^-^^11^ Recently, a systematic review reported considerably higher mean values of homocysteine and lower levels of vitamin B12 in vegetarians compared to omnivores.^12^ Because of absence of vitamin B12 in vegetables and it is indispensible coenzyme for homocysteine metabolism, all the vegetarians are at greater risk of having increased homocysteine level than are omnivores.^10^

A study in low income urban population in Karachi reported mild hyperhomocysteinemia with deficiency of folate, B12 and B6.^13^ There is scarcity of data about the measurement of Hcy level in vegetarians’ in our local population. Therefore, the current study was accomplished in the population of Mithi, District Tharparker (Sindh) where a significant proportion of individuals (Hindu community) adhere to a vegetarian diet throughout their lifespan and have never consumed animal products, except in the form of milk or milk products, due to family conventions or religious doctrines. 

The aim of present study was to investigate serum homocysteine levels in apparently healthy vegetarians and ominvores in Mithi, district Tharparker, Sindh, Pakistan.

## METHODS

This observational study was conducted in the Department of Biochemistry, BMSI, JPMC, Karachi in 2012. A total of 200 healthy volunteers (age ranging from 20-40 years) were recruited for this study, from the general population of Mithi, district Tharparkar, Sindh, Pakistan. Out of 200 subjects, 100 (50%) were vegetarians and 100 (50%) omnivores. Out of 200 subjects, 146 (73%) were males and 54 (27%) females. The vegetarian group had adhered to a vegetarian’s diet since their childhood. Except for the small consumption of dairy products, they ate no animal source food such as meat, poultry, fish, or eggs. Our all study subjects were apparently healthy, and had no physician-diagnosed disease (especially inflammatory disease). An informed consent was taken from all participants and a detailed questionnaire with all the clinical data was completed for each participant on the day of recruitment. This included information about the participant’s blood pressure, smoking habits, height and weight measurements, dietary status, milk consumption, etc. Individuals with following status were excluded from this study, those taking multi vitamins, diabetics, pregnant and lactating females, alcohol users, cigarette smokers, having gastrointestinal, autoimmune and chronic diseases.

Body weight was measured in kilograms with an electronic scale and standing height was measured in centimeters by stadiometer. BMI was calculated by using the formula:

weight (Kg)/height (m^2^).

Five milliliter blood was collected from each volunteer, after an overnight fasting. The serum was separated and stored at -70 ^0^C until analyzed for homocysteine. Homocysteine level was measured by the chemiluminescence enzyme immunoassay method by using immulite 1000.

All analyses were performed on SPSS version 17. Values of quantitative variable were presented by mean with standard deviation and comparison was done by applying Student t- test. The p value <0.05 was taken significant.

## RESULTS

**Fig.1:**
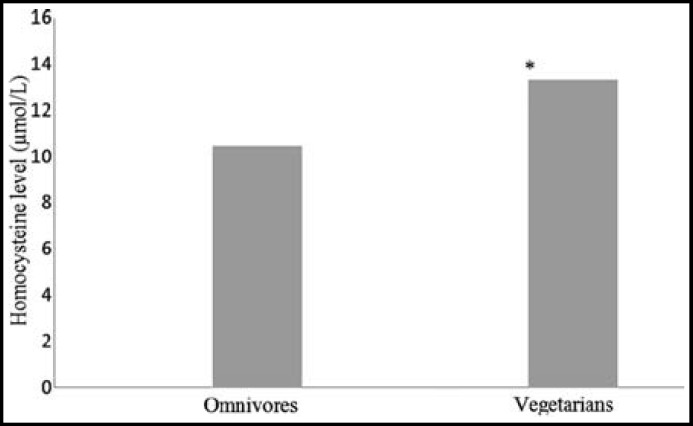
Serum homocysteine concentration in vegetarians and omnivores (*p<0.001)

Table-I shows the comparison of mean values of anthropometric parameters in both groups. Serum Hcy level was significantly higher (p<0.001) in vegetarian group compared to omnivores (Fig.1).

We further grouped and analyzed our study subjects according to their gender and according to Hcy level (greater than or lower than 15µmol/L). Out of 200 subjects, there were 146 male (vegetarian 74 and omnivores 72) and 54 females (vegetarian 26 and omnivores 28). A considerable higher number of vegetarian subjects 30 (30%) were having Hcy >15µmol/L compared to omnivores (6%) (p<0.001). Significantly higher number of omnivores subjects 94 (94%) were having Hcy <15µmol/L compared to vegetarians 70 (70%), (p<0.001), (Table-II). Gender-wise comparison showed that 27.02% male and 38.46% females had >15µmol/L serum Hcy level in vegetarian group and 6.9% male and 3.5% females had >15µmol/L serum Hcy level in omnivores group, but there was no considerable difference in the mean value of Hcy observed between male and females in both groups. No noteworthy difference was observed when male vegetarians were compared with female vegetarians in both groups (p>0.05) and there was no significant difference found when male omnivores compared with female omnivores in both groups (p>0.05), (Table-II).

## DISCUSSION

The elevated blood concentration of Hcy is a newly emerging independent reason for arthrosclerosis and coronary artery disease.^2^^-^^4^ In the current study, vegetarian have significantly lower body weight and body mass index (BMI), these results are similar with the several studies.^14^^-^^16^

Present study observed significantly higher serum Hcy level in vegetarians compared to omnivores. This result is in agreement with earlier studies on vegetarians, accomplished in several other countries.^9^^-^^12^^,^^17^^-^^18^ The main reason behind this significantly high level of Hcy in vegetarians is that the vegetables are poor source of vitamin B12 and it is essentially required for Hcy remethylation to methionine. Consequently, in vegetarians lack of vitamin B12 causes elevation in Hcy level.

It has been documented in the literature that vegetarians’ have considerably decreased serum levels of vitamin B12 compared to omnivorous. Several studies have reported considerably negative correlation between vitamin B12 and Hcy levels.^12^^,^^18^ Recently, a study in Bangladesh reported that mean value of vitamin B12 was considerably lower in vegetarian compared to omnivores group but they did not measured Hcy level.^19^

After dividing our subjects into two subgroups (having Hcy level higher than 15µmol/L and lower than 15µmol/L), we observed that vegetarian group had significantly higher value of Hcy than omnivores.

Our study showed that among the vegetarians 30% and omnivores 6% had elevated Hcy levels (p<0.001). While Elamadfa and Singer^20^ reported that in vegetarian 52% and non vegetarian 45% had high Hcy levels. Karebudak *et al*.,^10^ showed that among vegetarian 16.7% had elevated Hcy level. In contrast to ours and most of the others result documented in literature, a recent study found elevated levels of Hcy in both vegetarians and non-vegetarian.^21^ They described that it could be because of low folate intake in non-vegetarian and low vitamin B12 content in vegetarian population. 

Present study observed no significant gender-wise difference in Hcy level in both groups. This is similar to results demonstrated by Ganeshan et al.,.^21^ Conversely, previous several studies described elevated serum Hcy level in men compared to women.^22^^-^^23^ Refsum* et al*.,^24^ elucidated this difference is due to difference in muscle mass, hormone and vitamin status. Recently, a study in Pakistan reported serum Hcy levels >15 µmol/l in elderly women that indicates Hcy level increases with age. ^25^

There are few limitations to our study like, small sample size and measurement of only Hcy level. All the nutritionists because of its widely established good impacts on health recommend the vegetarian diet and it is also considered cardio-protective but only vegetarian diet has some bad effects like deficiency of vitamin B12 which is one of the most common causes of hyperhomocysteinemia. A study in urban population in Karachi, reported that the prudent diet (comprised of eggs, fish, vegetables, fruits) have a protective effect towards the development of hyperhomocysteinemia.^26^ Therefore, it is suggested to use mix diet or use vitamin B12 supplements with vegetarian diet to avoid consequences of lack of vitamin B12.

**Table-I:** Comparison of anthropometric variables among vegetarians and omnivores

**Anthropometric variables**	**Omnivores ** **(n=100)**	**Vegetarians ** **(n=100)**
Age (Yrs)	28.71±5.64	27.7±5.8
Height (Cm)	168±4.02	166±4.57
Weight (Kg)	69±5.21	61±7.14*
BMI (Kg/m²)	21±1.50	18±2.00*

Values are expressed as mean ±SD,

* p<0.05,

**Table-II:** Gender-wise comparison of serum homocysteine levels in vegetarians and omnivores subjects

**Variables **		**Homocysteine (>15µmol/L)**		**Homocysteine (<15µmol/L)**	
		**Vegetarian No (%)**	**Omnivores No (%)**	**Vegetarian No (%)**	**Omnivores** **No (%)**
Male	146				
-Vegetarian - Omnivores	74 72	20(27.02)	05(6.9)*	54(72.97)	67(93.5)*
Female	54				
-Vegetarian - Omnivores	26 28	10(38.46)┼	01(3.5)*+	16(61.53)ѱ	27(96.4) *+
Total		30 (30)	06(06)*∞	70(70)	94(94)∞

No= Number of subjects.

* p<0.01when male and female vegetarians’ compared with male and female omnivores in both groups.

┼ p=>0.05, when male vegetarians’ compared with female vegetarians’ in both groups.

+ p=>0.05 when male omnivores compared with female omnivores in both groups.

∞p=<0.001 when total vegetarians’ compared with total omnivores in both groups.

## CONCLUSION

The present study found higher Hcy level in vegetarians, which indicates that vegetarians are more prone to develop cardiovascular disease. Therefore, vegetarians should use extraneous sources of Vitamin B12 to prevent hyperhomocysteinemia and this step would reduce CVD risk.

## Authors’ Contribution:

**AK:** Designed the study, collected the samples, prepared the manuscript

**ZNA: **Supervised the study, and helped in drafting and revising the manuscript

**RMI: **Helped in drafting and revising the manuscript.

**MB: ** Analyzed the data and was involved in preparing and finalizing the manuscript. 

The final manuscript was approved by all authors for publication.

## Notes

***Source of funding:*** None.
